# An experimental study of the putative mechanism of a synthetic autonomous rotary DNA nanomotor

**DOI:** 10.1098/rsos.160767

**Published:** 2017-03-22

**Authors:** K. E. Dunn, M. C. Leake, A. J. M. Wollman, M. A. Trefzer, S. Johnson, A. M. Tyrrell

**Affiliations:** 1 Department of Electronics, University of York, Heslington, York YO10 5DD, UK; 2 Biological Physical Sciences Institute, Departments of Physics and Biology, University of York, Heslington, York YO10 5DD, UK

**Keywords:** molecular machine, DNA nanotechnology, rotary motor, strand displacement

## Abstract

DNA has been used to construct a wide variety of nanoscale molecular devices. Inspiration for such synthetic molecular machines is frequently drawn from protein motors, which are naturally occurring and ubiquitous. However, despite the fact that rotary motors such as ATP synthase and the bacterial flagellar motor play extremely important roles in nature, very few rotary devices have been constructed using DNA. This paper describes an experimental study of the putative mechanism of a rotary DNA nanomotor, which is based on strand displacement, the phenomenon that powers many synthetic linear DNA motors. Unlike other examples of rotary DNA machines, the device described here is designed to be capable of autonomous operation after it is triggered. The experimental results are consistent with operation of the motor as expected, and future work on an enhanced motor design may allow rotation to be observed at the single-molecule level. The rotary motor concept presented here has potential applications in molecular processing, DNA computing, biosensing and photonics.

## 1. Background

**Figure 1.**
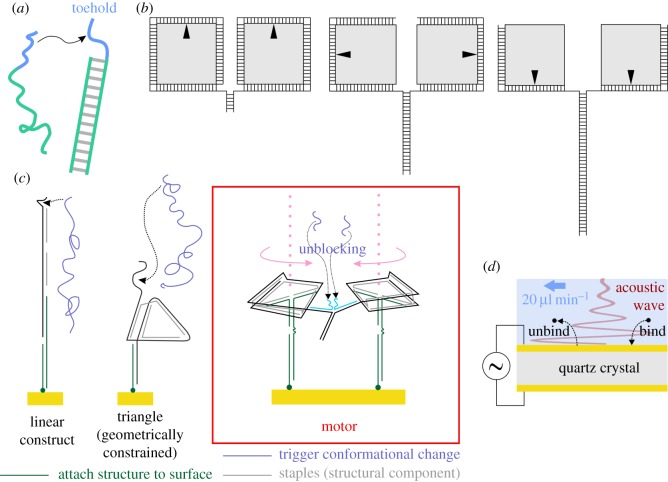
(*a*) Toehold-mediated strand displacement. An invader binds to the toehold (blue) and displaces the shorter incumbent via branch migration. (*b*) The underlying principle of the rotary motor concept described in this paper. The motor consists of two ‘wheels’ (grey squares); each wheel has a ‘tape’ wrapped round it. The two tapes are mainly complementary and are designed to displace each other from the wheels. This is intended to exert torque on the wheels, causing them to turn. (*c*) The DNA structures and devices studied in this paper. From left to right: linear construct, triangle, rotary motor (based on two squares). The motor is shown with the brake applied. The brake consists of a pair of blocking strands (bright blue) and it is released through displacement of the blocking strands by the unblocking strands (lilac). For clarity, parts of the motor that are irrelevant to the operating mechanism are not shown. The motor is illustrated in more detail in figure 4 and the electronic supplementary material. (*d*) Operation of a quartz crystal microbalance with dissipation monitoring. The piezoelectric crystal oscillates, driven by an applied voltage. The generated acoustic wave propagates into solution through a layer of surface-immobilized molecules. The frequency and energy dissipation of the wave reflect the mass and structure of the layer.

It was first suggested in the 1980s that DNA could be used to build nanostructures through self-assembly of oligonucleotides by complementary base pairing [1]. In a DNA nanostructure, the base sequence of each DNA strand is designed to ensure that it binds in the correct position and performs its intended function. Static DNA nanostructures described in the literature include polyhedra [2–4], tiles [5], solid blocks [6] and twisted or curved shapes [7]. DNA can also be used to make dynamic nanomachines that are capable of undergoing a change of state or conformation. Many such devices are driven by toehold-mediated strand displacement [8–10]. For this process, the initial state (figure 1*a*) consists of a double-stranded DNA construct with a short single-stranded toehold domain, and a single-stranded DNA invader that is complementary to the longer of the two strands in the main construct. The toehold-binding domain of the invader binds to the toehold, and then branch migration occurs. Eventually, the invading strand displaces the incumbent, leading to a final state that consists of a complete double helix and a shorter single-stranded molecule.

This exchange of strands can be used to operate molecular machinery, such as ‘DNA tweezers’ that open and close [11] and bipedal ‘walkers’ [12]. In some DNA motors, restriction enzymes are an integral part of the operating mechanism, and may be used in combination with strand displacement reactions to control molecular motion [13]. Strand displacement has been studied extensively in solution [8–10], and the effect of surface immobilization on the phenomenon has also been investigated [14].

The design of synthetic motors made from DNA is often inspired by their naturally occurring protein counterparts, such as kinesin. However, most synthetic DNA motors move linearly, rather than in a rotary manner, despite the presence in nature of highly efficient rotary machines such as ATP synthase [15]. Several non-autonomous DNA rotary motors have been presented, but these require external intervention to drive them from one state to the next. For instance, Yan *et al.* described a DNA machine consisting of a tile that underwent a conformational change involving rotation when particular strands were added [16], whereas Rajendran *et al.* presented a device in which the transition between right-handed and left-handed forms of DNA was used to induce rotation [17]. It has also been shown that it is possible to make a DNA rotary motor based on a catenane, a molecule consisting of interlocked rings [18].

In 2004, Tian and Mao presented a device that consisted of two counter-rotating wheels made from DNA [19], but this was not capable of autonomous operation, because DNA strands needed to be added to the reaction mixture in a particular order to drive rotation. By contrast, the machine presented here can rotate independently and does not require external intervention after the initial brake has been released.

In 2016, Ketterer *et al.* reported the synthesis of a ‘nanoscale rotary apparatus formed from tight-fitting three-dimensional DNA components’ [20]; in that work, the rotation was not driven and occurred at random simply as a result of Brownian motion. It has been suggested that coordinated opening and closing of hairpins could be used to drive an autonomous rotary DNA motor [21], but this idea has not been tested experimentally. In addition, the mechanism is likely to be affected significantly by unintended ‘leak’ reactions, processes that result from unintended molecular interactions and disrupt the operation of the system.

This paper describes the experimental study of the putative mechanism of an autonomous rotary DNA motor, through a series of experiments in which the underlying principles were tested and a prototype of the motor was examined. The motor is designed to be driven by sequential strand displacement reactions, and the concept is explained in the Methods section, in which the experimental procedures are also described. DNA molecules immobilized on a gold surface were studied using a quartz crystal microbalance with dissipation monitoring, and gel electrophoresis was used to provide complementary tests on the solution-phase motor. The experimental results were consistent with the motor operating as intended, but the methods used here did not allow rotation to be seen directly. The aims of the present study were: (i) to validate the hypothesis that sequential strand displacement reactions could catalyse significant structural rearrangement in a folded DNA construct on a surface; and (ii) to construct a rotary motor prototype based on this idea, confirming that it undergoes a structural transition as a result of strand displacement. Further experiments on an enhanced motor design may build on this work by imaging rotation with single-molecule resolution, and this is discussed towards the end of the paper.

## 2. Methods

### 2.1. Motor concept

The rotary motor mechanism described in this paper is based on two wheels that counter-rotate autonomously, driven by sequential strand displacement reactions in which complementary domains of two tapes become hybridized. This concept is illustrated in figure 1*b*. In its most general form, the motor features two ‘tapes’, each of which is wrapped around a ‘wheel’ and held in place by base pairing between complementary domains. The centres of the two wheels are at fixed positions, and the ends of the tapes are hybridized, such that the two wheels are connected via the tapes. The two tapes are mainly complementary, and the hybridized ends will subsequently act as a remote toehold [22] for the reaction in which the tapes mutually displace each other from the wheels. The tapes do not hybridize spontaneously owing to the presence of short non-complementary sections and associated blocking strands adjacent to the toeholds. This ‘brake’ mechanism is not shown explicitly in figure 1*b*, but is illustrated in figure 1*c*. When the brake is released, strand displacement can commence, through branch migration. As the tapes proceed to bind to each other, they unwrap themselves from the wheels. This causes torque to be exerted, and the wheels turn in opposite directions.

The joint through which a wheel is attached to a substrate must be able to accommodate rotation. To address this, the design features a polythymine linker, which is flexible and will not form significant secondary structure motifs. Despite the flexibility of this linker, it is desirable to avoid a situation in which the centres of the two wheels need to move closer together during rotation, as would occur if the tape were to be arranged in a flat spiral configuration. This problem can be eliminated by the use of three-dimensional wheels, where the diameter of the wheels does not change significantly during rotation. Here, the wheel is a multilayer structure, and tape is unrolled from one layer in each rotation.

**Figure 2.**
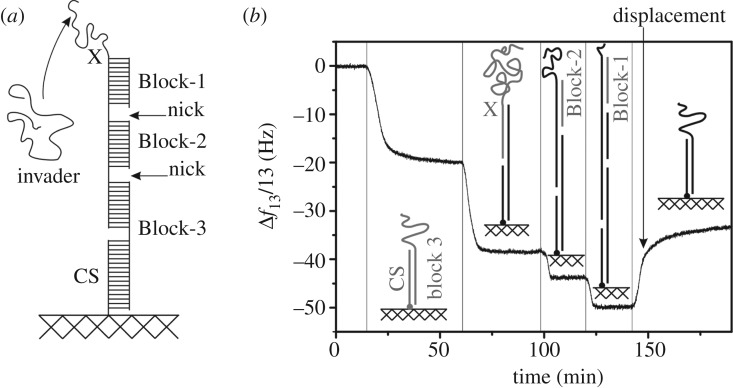
Sequential strand displacement in a linear complex. (*a*) Representation of the process to be examined, showing presence of nicks in the duplex backbone. CS is the capture strand. ‘Block’ strands block certain domains in X, where X is the target for the invader. The ‘Block’ strands are displaced from X by the invader, and Block-3 remains attached to CS at the end of the reaction. (*b*) QCM-D data for assembly of test construct and application of a single invader that displaces multiple strands. Sketches show the expected nature of the surface-immobilized molecules at the time the corresponding plateau is reached.

Inspection of the sketch in figure 1*b* reveals that the underlying mechanism of rotation in this motor is a series of strand displacement reactions. Consecutive domains of a long strand are displaced in order as branch migration occurs. To test this principle in isolation, the linear construct shown in figure 1*c* was examined. The reaction observed in this case differs from conventional strand displacement only in that there are two nicks in the molecule, where a nick is a break in one of the sugar–phosphate backbones of the duplex (figure 2*a*). To ascertain whether this reaction also occurred successfully in a molecule that was folded into a geometrically constrained configuration, the triangle structure (figure 1*c*, centre) was probed. The culmination of the work described in this paper was the study of the motor prototype shown in figure 1*c* (right-hand side).

The experimental techniques used in this investigation are described below.

### 2.2. Quartz crystal microbalance with dissipation monitoring

A quartz crystal microbalance with dissipation monitoring (QCM-D) [23] was used to study the behaviour of the DNA constructs. The QCM-D apparatus can measure small changes in the mass of a layer of surface-immobilized molecules and is based on a sensor consisting of a piezoelectric quartz crystal with a gold electrode on either side. Application of an AC voltage causes the crystal to oscillate, with the result that an acoustic wave propagates into the solution above it. The crystal is driven at its resonant frequencies or overtones, of which there are typically several. An increase in oscillation frequency indicates that mass has been removed from the crystal surface, while a decrease in frequency implies a mass increase. To measure the energy dissipated as the acoustic wave propagates through the molecular layer and into solution, the driving voltage is switched off and the decay of the amplitude is measured. The dissipation is related to the time constant of the decay. Generally, high dissipation implies a rather viscoelastic layer, while low dissipation implies that the layer is rigid. This paper focuses primarily on the frequency measurements, which mainly reflect changes in surface-immobilized mass. The dissipation measurements provide some limited additional insight.

For this investigation, all experiments were performed in a buffer of 1×TE with 1M NaCl. Unless otherwise stated, the pump flow rate was kept at 20 μ l min^−1^ except when the pump was switched off during sample changing. A Q-sense E4 system was used (best sensitivity 0.5 ng cm^−2^), with gold-coated sensors (QSX301, fundamental frequency 5 MHz), all supplied by Biolin Scientific. Sensors were cleaned immediately before each experiment using the procedure described in [14].

### 2.3. Gel electrophoresis

Agarose gel electrophoresis was performed using a gel containing 2% w/v agarose (Promega), with 1×TAE buffer (made from 10× stock), using a MultiSUB Midi gel kit from Scientific Laboratory Supplies with a Bio-Rad PowerPac Basic. Before cooling, SYBR Safe dye (Invitrogen) was added to the molten gel for staining and after running the gel was scanned with a Bio-Rad Chemidoc MP imager. The gel was run at 75 V for 45 min. Polyacrylamide gel electrophoresis was performed using a Bio-Rad precast gel (10%, TBE) and mini PROTEAN kit with 1×TBE buffer from Fisher Scientific. The gel was run at 100 V for 60 min on a laboratory bench, with two ice packs attached to the gel tank to prevent overheating. Staining was performed for a few minutes using SYBR Safe stain from Invitrogen (1×, diluted with ultrapure MilliQ water) and the gel was scanned using a Bio-Rad Chemidoc MP imager.

The two techniques used here are complementary—QCM-D provided time-resolved measurements of motors immobilized on a surface, whereas gel electrophoresis was used to examine the static configuration of the molecular machines before and after operation in solution. QCM-D is a very powerful technique and can be used to measure continuously for hours with very high time resolution. Both are ensemble measurements, and both are label-free, avoiding the question of whether the addition of a label could affect the behaviour of the device under investigation. QCM-D and gel electrophoresis both allow multiple samples to be studied in parallel, and only a minimal amount of prior information is needed to design the experiments, which is extremely useful for the study of a molecular machine that has never previously been tested. Little data processing is required for either experiment. Gel electrophoresis is well established in DNA nanotechnology, but QCM-D is less widely known in this area. Recent studies have suggested that QCM-D has considerable potential for the study of DNA structures and machines [14,24] and this work provides further evidence for this.

### 2.4. Materials

All chemicals were acquired from Sigma Aldrich unless otherwise stated. DNA oligonucleotides were purchased from Integrated DNA Technologies. They were resuspended in 1×TE, typically to a concentration of 100 μ*M*, and usually stored thereafter at −20^°^*C*. Sequences of individual strands and further details are given in the electronic supplementary material, together with the recipes used to prepare all of the samples.

## 3. Results and discussion

### 3.1. Sequential strand displacement in a linear complex

To test the hypothesis that a single long invader could displace multiple strands from an immobilized target complex, the system depicted in figure 2*a* was studied using QCM-D. The first stage of the experiment involved immobilization of a ‘capture complex’ via chemical bonds made between the gold surface and a thiol modification at one end of the complex. The capture complex itself comprised two strands, called CS and Block-3. As the capture complex was immobilized, the frequency decreased (figure 2*b*), owing to the increase in the mass attached to the sensor. In the second step, the DNA molecule X was added, where X had the correct sequence to bind to the capture complex, leaving a long single-stranded overhang, as illustrated. Next, two more DNA strands were added, and these bound to the overhang, leaving only a short single-stranded toehold. Further decreases in frequency were observed (figure 2*b*), corresponding to additional increases in mass. The final step was the addition of an invading strand, which bound to the toehold and displaced all three of the blocking strands from the long strand in the immobilized complex, resulting in loss of the strands X, Block-2 and Block-1 from the surface.

The frequency change observed during the displacement reaction was approximately 55% of that measured during binding of the strands X, Block-2 and Block-1. This implies that the sequential strand displacement process was not 100% efficient, as expected—surface-specific phenomena interfere with processes that occur in immobilized molecules, and decrease the efficiency of the reaction. However, the final baseline was higher than the value observed (at around 70–90 min) for the plateau corresponding to the construct comprising CS, Block-3 and X, which would not be possible if only Block-1 had been displaced from the immobilized constructs. The data were therefore consistent with the hypothesis that a single invader can displace multiple targets within the same surface-immobilized molecule, justifying the subsequent experiments and development of a motor based on this concept.

### 3.2. Sequential strand displacement in a folded structure

**Figure 3.**
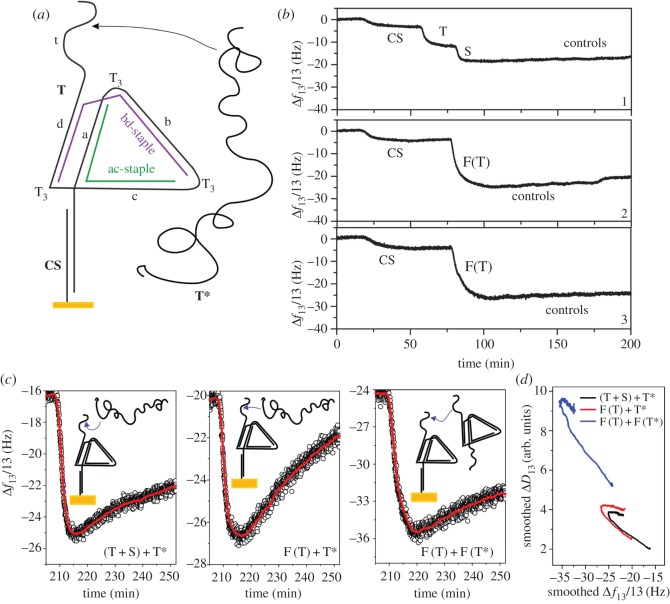
Observing strand displacement in a folded nanostructure. (*a*) Schematic diagram of the triangle structure. The long strand T had six domains (t, d, c, b, a, RC-CS), separated with T triplets where necessary. The long strand T was folded into a triangular conformation by means of two shorter strands (bd-staple) and (ac-staple) and the triangle could be immobilized through attachment to the CS strand. (*b*) QCM-D data which illustrate immobilization of strand CS and either isothermal on-surface assembly of triangles (*T*+*S*) or attachment of pre-folded triangles (F(T)). Control strands were used to test whether the triangle had assembled correctly. (*c*) Strand displacement in the nanotriangles. The immobilized triangles were exposed to unfolded or folded reverse complements, which bound to their targets and induced displacement. Red lines: smoothed data (50 point adjacent average filter). (*d*) Dissipation changes as a function of frequency changes for the time period shown in part (*c*). A 20 point adjacent-averaging smoothing filter was used.

QCM-D experiments were also performed on a geometrically constrained structure, formed by using two 23 nt oligonucleotides to fold a 73 nt oligonucleotide (T) into a triangular configuration (figure 3*a*). The shorter strands were named ‘staples’, following the convention of DNA origami [5]. Surface-immobilization of this structure was achieved via hybridization of the triangle strand with a pre-immobilized capture strand (CS), as shown in figure 3*a*.

Figure 3*b* presents the overtone-normalized frequency shifts observed with QCM-D during the assembly of a triangle *in situ* on the surface (in part 1) and immobilization of pre-folded triangles (in parts 2 and 3), followed by the application of control strands designed to bind to free domains in the immobilized structures. The bottom two panels of figure 3*b* show the application of the same molecules in parallel. The first phase of all three experiments performed was the immobilization of CS alone, corresponding to a frequency decrease of 3–5 Hz. The variation observed is attributable to uncontrollable minor differences between the sensors.

The top panel of figure 3*b* relates to the experiment in which the triangle was folded isothermally on the surface. After immobilization of CS, the strand T was supplied, leading to a decrease in frequency as T hybridized to CS. For rigid layers immobilized on the surface of a QCM-D sensor, the Sauerbrey equation (commonly used in analysis of QCM data [23]) predicts that the overtone-normalized frequency shift should be directly proportional to the change in the surface mass density. However, it will be seen that *Δf*_13_/13 did not scale with the molecular mass of the oligonucleotides added; the strand T contains 73 nt and its addition induced a shift of approximately 8.5 Hz, which is just over half of what would be expected based on the shift of approximately 3.5 Hz observed for the 16 nt strand CS. This implies either that not all of the CS oligonucleotides captured a T or that the Sauerbrey equation does not apply here, which would be unsurprising owing to the non-rigid nature of the surface-immobilized layer of biomolecules.

The addition of the staple strands resulted in a further frequency decrease, corresponding to an additional increase in immobilized mass. This was associated with an increase in dissipation, indicative of conformational change. Control experiments (described below) suggested that no triangle domains were left single-stranded and no staples were half-bound, consistent with proper folding of the triangle. These results are interesting in their own right because they suggest that it is possible to assemble a complex structure on the surface isothermally. It is likely that the staples cooperate during folding, because the binding of one staple will bring into closer proximity the binding sites for the next, which will then be able to bind more easily [25,26].

After folding, the dissipation values were comparatively high, approaching two units for the CS-T complex, and this implies that the Sauerbrey equation should not be used here because the layer is viscoelastic rather than rigid [27]. In fact, the shift in frequency resulting from addition to the staples was higher than would be predicted from the Sauerbrey equation. If a linear relationship existed between frequency changes and mass, the results would imply that 1.25 copies of each staple had bound to each T molecule, and because that is impossible, this result represents a further indication that the Sauerbrey equation does not apply in this regime. This is to be expected for a layer of biological molecules, which have a complex three-dimensional structure and dissipate acoustic energy, displaying viscoelastic behaviour.

The data presented in the bottom two panels of figure 3*b* show immobilization of a pre-folded triangle by hybridization with CS. The frequency shift will be seen to be greater in magnitude than the combined shifts induced by application of T and S in the top panel, suggesting that the use of pre-folded triangles may lead to a higher-density molecular layer.

In all cases, it was necessary to test whether the triangle structures had folded completely. Two primary folding ‘errors’ are conceivable. Either a staple could be ‘half-bound’, where one domain is attached to the triangle and the other is single-stranded, or it could be missing completely. In all three experiments, to test for these errors, control strands were applied after the supposed formation of a surface-immobilized layer of folded triangles (figure 3*b*, all graphs, section marked ‘controls’). The sensor was supplied in turn with a series of control samples containing strands that should either bind to a free domain of a staple, or a free domain of the T strand. In all cases, minimal frequency shifts were observed, attributable to changes in the mixture of molecules passing over the sensors rather than the binding of any strand to the surface-immobilized constructs. This suggested that all structures were indeed completely folded, including the triangle folded isothermally *in situ* on the surface. Use of the online analysis package NUPACK [28] confirmed that 96% or more of the control strands should hybridize to their target domains, if available, and although this simulation uses the energy parameters established for solution-phase reactions, it provides a good indication that this control experiment is sound.

Strand T* was complementary to strand T, and the exposure of folded triangles to T* resulted in some unfolding of T (figure 3*c*, left and centre panels). The data show a drop in frequency that corresponds to binding of T*, followed by an increase as staples are displaced and T unfolds. The same effect was observed for the application of T* strands that had been folded up into triangles in the same way as T. In all cases, the loss of mass resulting from unfolding was significant although comparatively small, and the timescale was long (many tens of minutes). The amount of mass lost appeared to vary between the three cases.

The data presented here do indicate that strand displacement is possible in a structure that is geometrically constrained, an important result for the rotary motor concept. However, the results also suggest that the efficiency of the reaction can be quite low. This may be associated with molecular crowding effects, which are known to have an effect on strand displacement on surfaces [14]. Importantly, it was necessary for T* to displace at least three staple domains before any staples could be lost, and this means that at least some of the triangles were partly or entirely unfolded by T*. It would not have been possible for displacement of any domain to be initiated prematurely by the interaction between AAA and TTT domains in the T and T* strands, because AT-rich toeholds 3 nt in length do not permit displacement in surface-immobilized DNA machines [14].

Figure 3*d* shows that dissipation and frequency changes are not perfectly correlated. This is an interesting result because it implies that the structure of the molecules is changing independently of the changes in mass, which is exactly what would be expected from unfolding of triangles. It has been demonstrated that the acoustic ratio (*ΔD*/*Δf*) can be used as a measure of the conformation of DNA molecules on surfaces [24], but it is difficult to use this *a priori* without any reference structures.

### 3.3. Rotary motor

**Figure 4.**
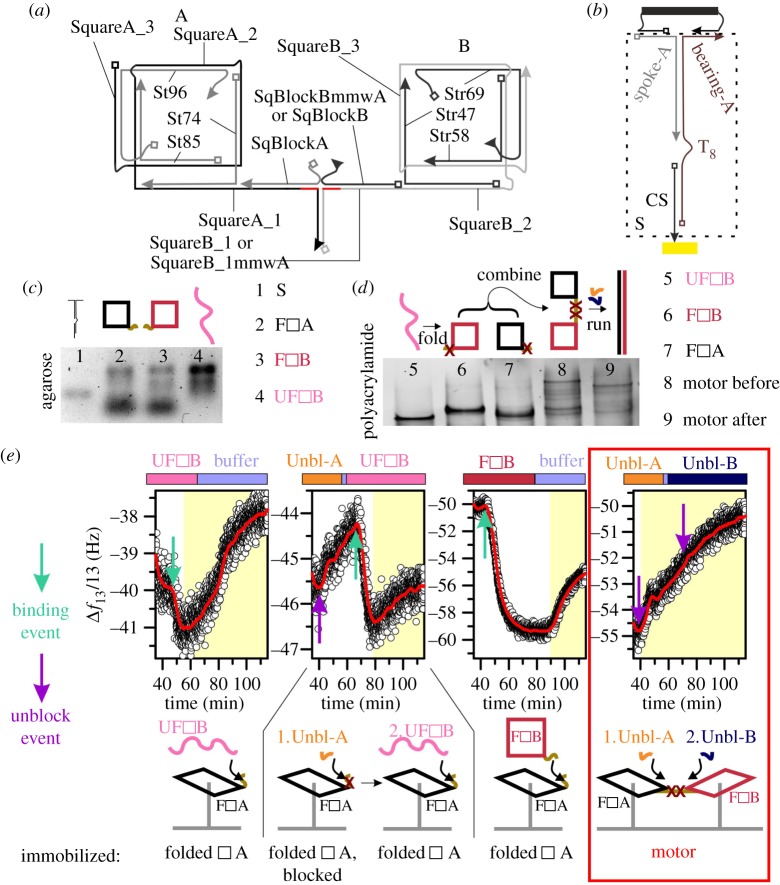
The rotary motor. (*a*) Schematic diagram of the motor which shows how it was constructed. The staple strand names begin with ‘St’ or ‘Str’. The tape for square A is made from three strands (SquareA_1, SquareA_2 and SquareA_3) as is the tape for square B (SquareB_1 or SquareB_1mmwA, SquareB_2 and SquareB_3). In each square, these strands are connected through connection domains (not shown here) that protrude like spokes from the motor. As described in the text, the connection domains are irrelevant to the motor mechanism because they remain inert throughout operation, but they are shown explicitly in the electronic supplementary material. Apart from the connection domains and the domains marked in red, the tapes are complementary to each other. (*b*) The structure used to immobilize a square. (*c*) Agarose gel which shows the assembly of the surface connector illustrated in (*b*), the two folded squares and an unfolded square (made from only the tape strands, without any staples). (*d*) Polyacrylamide gel which shows the most important bands for the indicated samples: unfolded square of type B, folded squares of both types and assembled motor—‘before’ and ‘after’ operation. (*e*) QCM-D data which show strand displacement in motor components or the operation of the motor. Note that the graphs have different *y*-axis scales but all show the same time window. The labels at the bottom of the figure show what structure was immobilized on the surface, and the coloured bands above the plots indicate what was applied to the sensors, where ‘Unbl’ refers to an unblocking strand. The sections of the plot shaded in yellow correspond to mass loss from the surface, resulting from displacement. Binding and unblocking events are marked. Red lines: smoothed data (100-point adjacent average filter). (*c*–*e*) Samples are described using simplified sketches and abbreviations—F denotes ‘folded’, U denotes ‘unfolded’, open square denotes ‘square’, ‘Unbl’ denotes ‘unblocking strand’.

The rotary motor prototype is shown in figures 1*c* and 4*a*. It was assembled in stages in solution from a number of oligonucleotides, as described in the electronic supplementary material. It consisted of two squares (A and B), made from ‘tape’ strands and staples that held the tape strands in place. The design principles are the same as for the triangle presented in the previous section. The two squares were linked, as shown. The domains adjacent to the linked sections were not complementary and were hybridized to blocking strands. Release of the blocking strands allowed the hybridized domains to act as a remote toehold for initiation of strand displacement, driving rotation of the motor. Each tape actually consisted of a series of three oligonucleotides, joined together by hybridization of connecting domains that protruded from the wheels like spokes. For clarity, these spokes are not shown in the figures because they are irrelevant to the reaction mechanism, but they are illustrated in the electronic supplementary material. The spokes remained double-stranded and did not change partners throughout motor operation. They were not expected to interfere with rotation because each spoke could pass over or around the other wheel.

The squares were immobilized on a surface via the structure shown in figure 4*b*, which includes the thiolated strand CS. The assembly of the tapes from their constituent parts, the surface-immobilization connector and the squares themselves was confirmed prior to immobilization using agarose gel electrophoresis (figure 4*c*). It was not feasible to test assembly using QCM-D in the same way as for the triangle, because, in this case, the incorporation of one of the staples was necessary for immobilization, and the greater complexity of the design would have significantly increased the required number of control strands. The surface-immobilization connector produces one clear band in the gel, which indicates that this structure has assembled correctly. Similarly, there is one dominant band in the lane corresponding to the unfolded square (assembled tape), although some smearing of the band is apparent.

The squares were folded as described in the electronic supplementary material. The staples were present in excess, which means that the presence of the lower band in the lanes marked F□A and F□B does not imply poor assembly. However, it is not possible to distinguish between the unfolded and folded constructs using agarose gel electrophoresis, and a polyacrylamide gel was therefore used to provide higher resolution (figure 4*d*), showing both assembly and operation of the motor. In this gel, there is a distinct difference between the position of the band identified as unfolded square B (lane 5), and that identified as folded square B (lane 6), indicating that folding was successful. Folded square A (lane 7) runs slightly further than folded square B, owing to the asymmetry in the positions of the spokes mentioned above. Lane 8 shows the motor before operation, obtained by hybridization of folded square A and folded square B. At this stage, the brake of the motor was applied, which means that the blocking strands were attached, preventing the unrolling of the tapes. The highest molecular weight band in lane 8 is postulated to be the assembled motor. Some of the squares did not connect with another square. Lane 9 shows the motor after incubation with the unblocking strands, which was intended to remove ‘SqBlockA’ and ‘SqBlockBmmwA’ by displacement. This was expected to release the brake, allowing strand displacement to proceed. Comparing lanes 8 and 9 reveals that the unblocking process induced a significant structural rearrangement in the motor, which is the expected result.

However, gel electrophoresis is not conclusive and provided information only on static structures. By contrast, the subsequent QCM-D experiments allowed structural changes to be measured in real time. In addition to probing the operation of the motor, these measurements provided a further indication that folding had been successful, because the observation of any changes corresponding to operation of the motor or unfolding of squares would have been highly unlikely if folding had been incomplete.

QCM-D experiments were performed to investigate motor operation in more detail. In contrast to the previous QCM-D measurements, DNA was co-immobilized with the backfilling agent mercaptohexanol, which competes with the thiolated DNA for access to the surface. The aim of this was to reduce the density of machines on the surface and consequently decrease any inhibitory effects arising from intermolecular interactions. The results of the QCM-D experiments are shown in figure 4*e*.

The first panel shows the effect of supplying an unfolded square of type B to an immobilized square of type A. This is directly analogous to the experiments in which T* was applied to folded triangles. In the same way, the binding of the unfolded square gave rise to a decrease in frequency. This was followed shortly thereafter by a slow increase, suggesting that the immobilized square unfolded and lost mass.

The second panel shows the result of a similar experiment, but in this case the immobilized square was initially blocked with a blocking strand, and was unblocked before the unfolded square was applied. Removal of the blocking strand is expected to cause a small reduction in mass, and the data do show a corresponding frequency increase. The result of applying the unfolded square was qualitatively similar to the case in which the immobilized square had not been blocked, but the frequency shift corresponding to unfolding was small. This may be attributable to incomplete unblocking in a surface-immobilized molecular layer consisting of only one type of square.

To investigate whether one folded square could induce unfolding in another, a fully folded square of type B was applied to an immobilized folded square of type A. This resulted in a frequency decrease, followed by a significantly delayed frequency increase. The former was attributable to binding of the square, whereas the latter was consistent with unfolding of the squares and release of staples. These experiments indicated that strand displacement reactions occurred as intended in the square constructs.

The full motor was assembled by the connection of two squares, and immobilized on the surface with mercaptohexanol. Unblocking strands were supplied to release the brake, and a slow frequency increase was observed thereafter, as expected to result from strand displacement, rotation and release of waste strands. This result is therefore consistent with the correct operation of the motor on the surface. The data suggest that rotation is initiated before the second unblocking strand has been supplied.

Alternative displacement pathways may exist, and it is not clear what effect this could have on the operation of the motor. It is possible that these pathways could be eliminated with an alternative motor design based on components made using DNA origami methods, as discussed below.

### 3.4. Further work

For further study, single-molecule biophysical experiments could be used to directly observe the dynamic functional operation of prototype rotary machines, but these measurements are beyond the scope of this paper, which represents a proof-of-concept study. Further work on the motor would comprise the development of an enhanced model (as described below), which would enable the use of techniques such as atomic force microscopy to visualize intermediate states. Direct tracking of rotation could be achieved using time-resolved super-resolution microscopy [29], which would involve tracking the position of a single fluorescent molecule (or group of them). Single-molecule fluorescence microscopy on this system poses significant challenges, such as: attaching the fluorescent label to the motor, compensating for microscope drift over the long timescale of this experiment, correcting for two-dimensional diffusion of the motor at the end of its tether and avoidance of excessive fluorophore photobleaching on the timescale of the rotation. Pursuit of such challenging single-molecule experiments could form the basis of a subsequent study, following on from the work presented here.

In future studies, the design of the rotary motor could also be enhanced, in three main respects. First, in the present implementation, the motor is only capable of performing 1.5 rotations, and this could be increased by a straightforward extension of the design. Second, the operation of the motor leads to disassembly of the motor, and the structure remaining on the surface is comparatively insubstantial. Third, it would be difficult to use the existing design in a single-molecule experiment because it is difficult to identify where a fluorescent label could be placed. These last two issues could be addressed simultaneously by constructing the wheels of the motor using DNA origami, a very popular technique for assembling DNA nanostructures [5,30,31]. Recent studies have explored the DNA origami folding process [25,26,32,33], and the results will allow more sophisticated origami structures to be assembled [34], including devices such as the enhanced motor suggested here. Alternatively, other DNA nanostructure assembly methods could be used [35–38].

## 4. Conclusion

This paper has described an experimental study of the putative mechanism of a prototype synthetic rotary motor made from DNA. The motor was designed to be driven by strand displacement and to be capable of autonomous operation after the brake was released. Results were also reported from experiments in which the phenomenon underlying the motor mechanism was investigated extensively, building on previous studies of strand displacement in surface-immobilized DNA nanomachines.

Ensembles of DNA rotary motors were examined using two complementary techniques: gel electrophoresis and QCM-D. The former was used to probe static motors in solution before and after operation, whereas the latter provided time-resolved data on surface-immobilized motors. Single-molecule measurements were beyond the scope of this study, but future work on an enhanced rotary motor design could involve a combination of the biophysical approaches of single-molecule fluorescence microscopy and structural imaging methods such as atomic force microscopy and cryoelectron microscopy. For the investigation described here, QCM-D and gel electrophoresis had a number of advantages.

The data in this paper represent promising experimental results that were consistent with the operation of the rotary motor as designed, and further work could enable the properties of the motor or an enhanced model to be exploited for real-world applications, such as manipulation of molecules, translocation of cargo through a nanopore, nanoswimmer propulsion, biosensors, hybrid photonic-biomolecular systems [39], and biomolecular computing.

## Supplementary Material

Supplementary Information: An experimental study of the putative mechanism of a synthetic autonomous rotary DNA nanomotor This file contains DNA sequences, detailed experimental procedures, results of NUPACK simulations and additional schematic diagrams of the motor.

## Data accessibility

The experimental data supporting this paper have been deposited in the Dryad repository and will be available after publication at the following address: http://dx.doi.org/10.5061/dryad.sj179 [40].
