# Supplementary material for: An experimental study of the putative mechanism of a synthetic autonomous rotary DNA nanomotor
Source: R Soc Open Sci. 2017 Mar 22;4(3):160767. doi: 10.1098/rsos.160767 (PMC5383820; doi:10.1098/rsos.160767)
Supplement: Supplementary Information: An experimental study of the putative mechanism of a synthetic autonomous rotary DNA nanomotor This file contains DNA sequences, detailed experimental procedures, results of NUPACK simulations and additional schematic diagrams of the motor. [file rsos160767supp1.pdf]

Supplementary Information: An experimental study of the putative mechanism of a synthetic autonomous rotary DNA nanomotor

K.E. Dunn*[1], M.C. Leake[2], A.J.M. Wollman[2], M.A. Trefzer[1], S. Johnson[1], and A.M. Tyrrell[1]

[1]Department of Electronics, University of York, Heslington, York, YO10 5DD, UK

[2]Biological Physical Sciences Institute, Departments of Physics and Biology, University of York, Heslington, York, YO10 5DD, UK

1 Sequences

The sequences of all DNA strands used in this work are given in the following tables. All sequences are written 5' to 3'. So-called 'pseudosequences' were used to design the molecules and represent their structure in terms of domains. For example, the 'pseudosequence' (CSr,T_8,BDA,X1r) denotes a sequence comprising, in order, the reverse complement of CS, 8 thymine bases, the domain called 'BDA', and the reverse complement of X1. For the simpler experiments, the symbol * is used to denote the reverse complement of a domain or strand, such as CS*. The sequences of CSr and CS* are identical. All strands were acquired

*To whom correspondence should be addressed. E-mail: katherine.dunn@york.ac.uk

with standard desalting purification, with the exception of the thiol-modified strand CS, for which HPLC purification was selected. All oligonucleotides were supplied by Integrated DNA Technologies.

1.1 Sequential strand displacement in a linear construct

The strands used to study sequential strand displacement in a linear construct are below. Note that the strand CS was used throughout this work. It is listed here only in this table.

**Name**	**Sequence**
CS	`ACA CGC ATA CAC CCA T-(thiol)`
X	`AAA CGC ACT ACG GCT CAG ATG TCC ACG AAC GCC ACC AAG G`
X*	`CCT TGG TGG CGT TCG TGG ACA TCT GAG CCG TAG TGC GTT T`
Block-1	`CTG AGC CGT AG`
Block-2	`TTC GTG GAC AT`
Block-3	`ATG GGT GTA TGC GTG TCC TTG GTG GCG`

Table 1: Sequences of DNA oligonucleotides used to examine sequential strand displacement in an immobilized linear construct.

1.2 Strand displacement in a geometrically constrained structure (‘Triangle’ experiment)

Table 2 provides the sequences used to assemble the triangle and trigger strand displacement reaction within it. Lower case letters represent domain names and the sequence T_3 is inserted in places to ensure flexibility at corners. The strand name T is not to be confused with the representation of a single thymine.

**Name**	**Pseudosequence**	**Base sequence**
T	CS*, a, T_3, b, T_3, c, T_3, d, t	ATG GGT GTA TGC GTG TTG TTG GTG TGT TTC TCC TCC TCC TTT CTG CCT GCC TTT TGC CGC CGC CGA TCA GCA T
ac-staple	a*, T_3, c*	CAC ACC AAC ATT TAG GCA GGC AG
bd-staple	b*, T_3, d*	GGA GGA GGA GTT TCG GCG GCG GC
Control-a	a_*	ACA CCA AC
Control-b	b_*	GAG GAG GA
Control-c	c_*	GGC AGG CA
Control-d	d_*	GGC GGC GG
T*	(CS*, a, T_3, b, T_3, c, T_3, d, t)*	ATG CTG ATC GGC GGC GGC AAA AGG CAG GCA GAA AGG AGG AGG AGA AAC ACA CCA ACA ACA CGC ATA CAC CCA T
RC(ac-staple)	(a*, T_3, c*)*	CTG CCT GCC TAA ATG TTG GTG TG
RC(bd-staple)	(b*, T_3, d*)*	GCC GCC GCC GAA ACT CCT CCT CC

Table 2: Sequences of DNA oligonucleotides used to construct the nanotriangles, confirm whether they had assembled correctly, and induce strand displacement. The abbreviation ‘t’ denotes the toehold. The _ subscript in the pseudosequences for the control strands indicates that the first and last nucleotide of these domains was omitted.

1.3 The rotary motor

Three tables are provided, listing the pseudosequence of each strand (Table 3), the sequence of each domain referred to in the pseudosequences (Table 4) and the final sequence of each strand (Table 5). The definition of the domains in the pseudosequences is given in Fig. S1, while Fig. S2 shows the strand exchange mechanism for the first quarter turn.

**Strand name**	**Pseudosequence**
SquareB_1	S1r,S2b,S3r,T2,conn_1
SquareB_1mmwA	S1r,S2bmmwA,S3r,T2,conn_1
SquareB_2	conn_1r,S4r,T2,S5r,T2,S6r,T2,conn_2
SquareB_3	conn_2r,S7r,T2,S8r,T2,S9r
SquareA_3	S9,A2,S8,A2,conn_3
SquareA_2	conn_3r,S7,A2,S6,A2,S5,A2,conn_4
SquareA_1	conn_4r,S4,S3,S2a,S1
SqBlockA	thA,S2ar,S3r
SqBlockB	S3,S2br,thB
SqUnblockA	(SqBlockA)r
SqUnblockB	(SqBlockB)r
Bearing-A	CSr,T8,BDA,X1r
Spoke-A	X2r,BDAr
Bearing-B	CSr,T8,BDB,X4r
Spoke-B	X3r,BDBr
St74	S7r,S4r
St96	X2,T2,S9r,S6r,T2,X1
St85	S8r,S5r
Str47	S4,S7
Str69	X3,T2,S6,S9,T2,X4
Str-58	S5,S8

Table 3: Pseudosequences of strands used to design the rotary motor.

**Domain name**	**Sequence**
S1r	CCA CGA TGC C
S2b	ACC TCA TCC C
S2bmmwA	GTT CAA TCC C
S3r	GAA TGC ATG A
S4r	CAT GAC GTT A
S5r	CCA TTG GCT G
S6r	ATG TGA CTG G
S7r	GAA TCT CAC T
S8r	GTT CGG CTC C
S9r	AAG TCA GTA T
S1	GGC ATC GTG G
S2a	ACC GAG TGG T
S3	TCA TGC ATT C
S4	TAA CGT CAT G
S5	CAG CCA ATG G
S6	CCA GTC ACA T
S7	AGT GAG ATT C
S8	GGA GCC GAA C
S9	ATA CTG ACT T
thA	ACC ACC
thB	GAG AGA
BDA	TCT ACC TAT T
BDB	CCA GTT GCT C
X1	CTA TAA CCG A
X2	ATC AAA GGG T
X3	AGC GAT TCA C
X4	ATT ACA AAA T
thAr	GGT GGT
thBr	TCT CTC
BDAr	AAT AGG TAG A
BDBr	GAG CAA CTG G
X1r	TCG GTT ATA G
X2r	ACC CTT TGA T
X3r	GTG AAT CGC T
X4r	ATT TTG TAA T
conn_1	GAG AAG AGA AGA GAA GAG AA
conn_2	GCA AAA CGA AAA GCA AAA CG
conn_3	GAA GAA GAA GAA GAA GAA GA
conn_4	GAA AGA AAG AAA GAA AGA AA
conn_1r	TTC TCT TCT CTT CTC TTC TC
conn_2r	CGT TTT GCT TTT CGT TTT GC
conn_3r	TCT TCT TCT TCT TCT TCT TC
conn_4r	TTT CTT TCT TTC TTT CTT TC

Table 4: Sequences of the individual domains of the oligonucleotides from which the rotary motor was assembled. Names refer to the pseudosequences defined previously.

Strand name	Sequence
SquareB_1	CCA CGA TGC CAC CTC ATC CCG AAT GCA TGA TTG AGA AGA GAA GAG AAG AGA A
SquareB_1_mmwA	CCA CGA TGC CGT TCA ATC CCG AAT GCA TGA TTG AGA AGA GAA GAG AAG AGA A
SquareB_2	TTC TCT TCT CTT CTC TTC TCC ATG ACG TTA TTC CAT TGG CTG TTA TGT GAC TGG TTG CAA AAC GAA AAG CAA AAC G
SquareB_3	CGT TTT GCT TTT CGT TTT GCG AAT CTC ACT TTG TTC GGC TCC TTA AGT CAG TAT
SquareA_3	ATA CTG ACT TAA GGA GCC GAA CAA GAA GAA GAA GAA GAA GAA GA
SquareA_2	TCT TCT TCT TCT TCT TCT TCA GTG AGA TTC AAC CAG TCA CAT AAC AGC CAA TGG AAG AAA GAA AGA AAG AAA GAA A
SquareA_1	TTT CTT TCT TTC TTT CTT TCT AAC GTC ATG TCA TGC ATT CAC CGA GTG GTG GCA TCG TGG
SqBlockA	ACC ACC ACC ACT CGG TGA ATG CAT GA
SqBlockB	TCA TGC ATT CGG GAT GAG GTG AGA GA
SqUnblockA	TCA TGC ATT CAC CGA GTG GTG GTG GT
SqBlockB_mmwA	TCA TGC ATT CGG GAT TGA ACG AGA GA
SqUnblockB_mmwA	TCT CTC GTT CAA TCC CGA ATG CAT GA
SqUnblockB	TCT CTC ACC TCA TCC CGA ATG CAT GA
Bearing-A	ATG GGT GTA TGC GTG TTT TTT TTT TCT ACC TAT TTC GGT TAT AG
Spoke-A	ACC CTT TGA TAA TAG GTA GA
Bearing-B	ATG GGT GTA TGC GTG TTT TTT TTT CCA GTT GCT CAT TTT GTA AT
Spoke-B	GTG AAT CGC TGA GCA ACT GG
St74	GAA TCT CAC TCA TGA CGT TA
St96	ATC AAA GGG TTT AAG TCA GTA TAT GTG ACT GGT TCT ATA ACC GA
St85	GTT CGG CTC CCC ATT GGC TG
Str47	TAA CGT CAT GAG TGA GAT TC
Str69	AGC GAT TCA CTT CCA GTC ACA TAT ACT GAC TTT TAT TAC AAA AT
Str58	CAG CCA ATG GGG AGC CGA AC

Table 5: Sequences of the DNA oligonucleotides used to assemble and unblock the rotary motor.

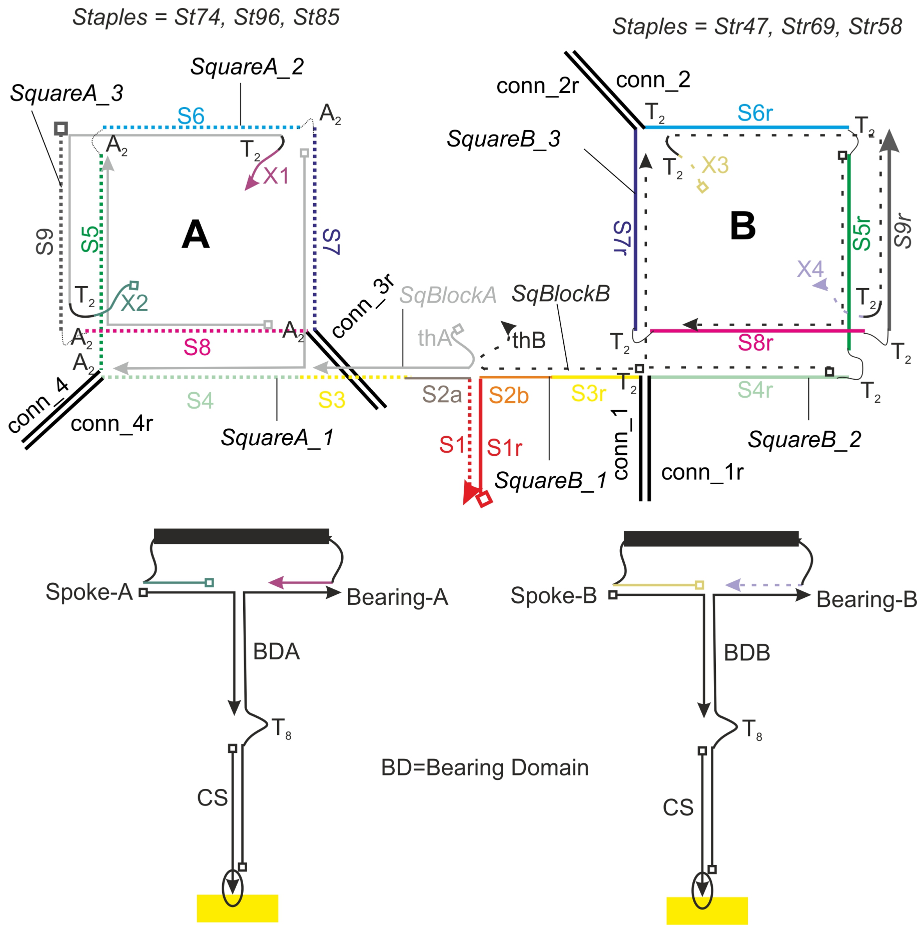

Fig. S1: A detailed schematic diagram of the motor components, showing the different domains. Strand names are given in italics, domain names are given in Roman type. All staple domains except X1-4 are left unlabelled. The double-stranded spokes formed by domains whose names begin with ‘conn’ play no part in the operation of the motor. For this prototype, the tapes were constructed using multiple oligos joined together, and the connection domains are irrelevant to the rotation mechanism.

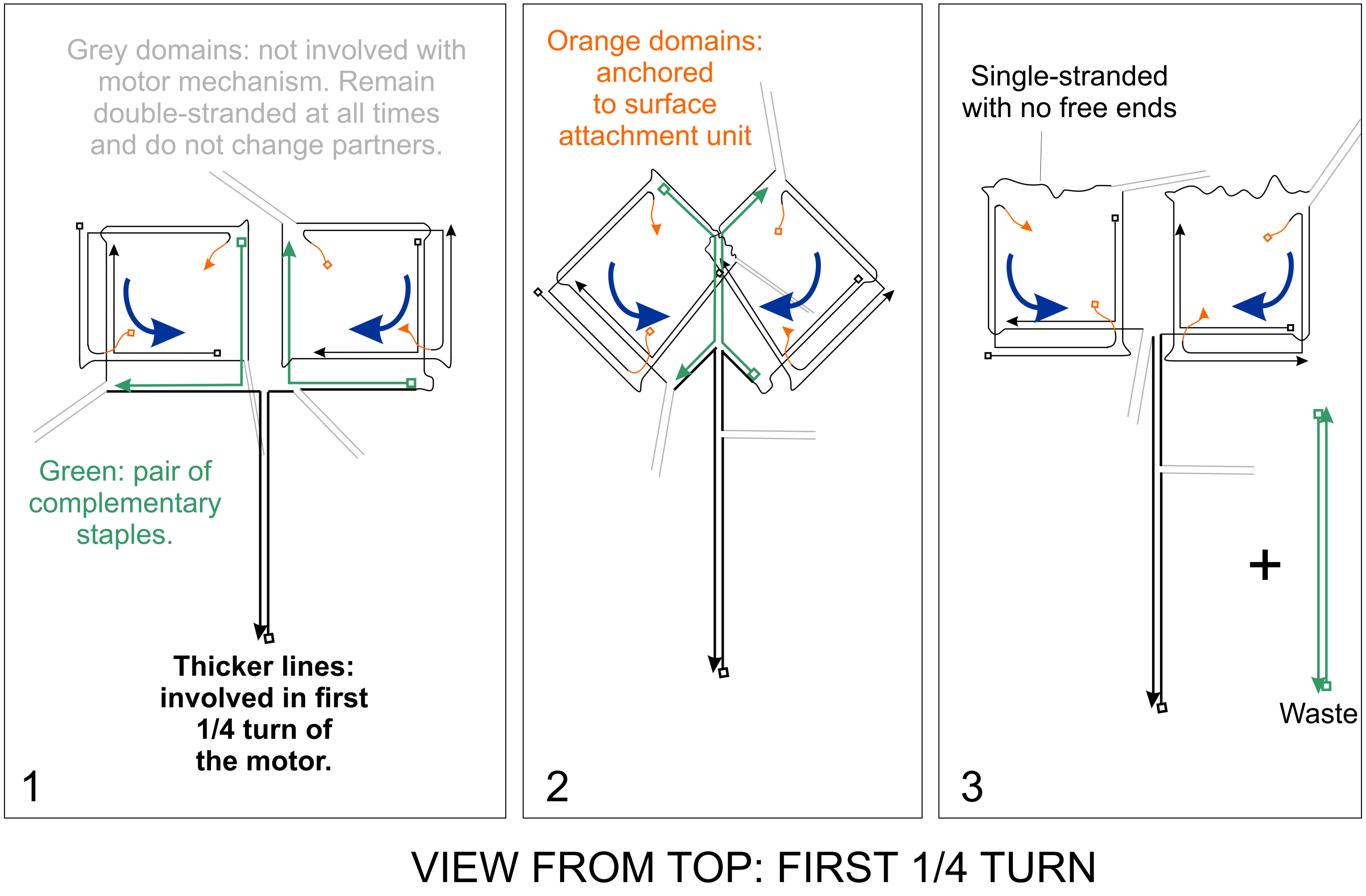

Fig. S2: Sketches showing the mechanism of strand exchange for the first quarter of a turn.

1.3.1 Analysis of motor domains using NUPACK

For data entry into NUPACK (see citation in main paper), each of the relevant domains was given a number, as listed in Table 6. Results are shown in Table 7.

Domain name	Strand number	Target number
S1r	1	11
S2bmmwA	2	3
S2bmmwAr	3	2
S3r	4	14
S4r	5	15
S5r	6	16
S6r	7	17
S7r	8	18
S8r	9	19
S9r	10	20
S1	11	1
S2a	12	13
s2Ar	13	12
S3	14	4
S4	15	5
S5	16	6
S6	17	7
S7	18	8
S8	19	9
S9	20	10
conn_1	21	25
conn_ 2	22	26
conn_ 3	23	27
conn_4	24	28
conn_1r	25	21
conn_2r	26	22
conn_3r	27	23
conn_4r	28	24
X1r	29	33
X2r	30	34
X3r	31	35
X4r	32	36
X1	33	29
X2	34	30
X3	35	31

Table 6: Numbering the domains for data entry into NUPACK.

**Complex**	**Description**	**Correct target?**	**Yield (%)**	ΔG **(kJ/mol)**	$\Delta G/(k_{\mathrm{B}}T)$
22-26	Dimer	Yes	100.0	-142.9	-58.7
21-25	Dimer	Yes	100.0	-129.5	-53.2
23-27	Dimer	Yes	100.0	-128.7	-52.8
24-28	Dimer	Yes	100.0	-122.2	-50.2
9-19	Dimer	Yes	99.9	-77.1	-31.6
1-11	Dimer	Yes	99.8	-78.4	-32.2
31-35	Dimer	Yes	99.5	-72.5	-29.8
7-17	Dimer	Yes	99.5	-69.4	-28.5
6-16	Dimer	Yes	99.3	-73.1	-30.0
5-15	Dimer	Yes	99.2	-68.5	-28.1
4-14	Dimer	Yes	99.2	-68.1	-27.9
2-3	Dimer	Yes	99.1	-66.7	-27.4
30-34	Dimer	Yes	98.8	-67.9	-27.9
29-33	Dimer	Yes	98.6	-64.5	-26.5
8-18	Dimer	Yes	97.9	-64.4	-26.4
12-13	Dimer	Yes	97.4	-78.4	-32.2
10-20	Dimer	Yes	97.3	-61.3	-25.1
32-36	Dimer	Yes	81.7	-55.0	-22.6
32	Monomer	Not hybridized	18.3	-1.6	-0.6
36	Monomer	Not hybridized	18.3	-2.2	-0.9
20	Monomer	Not hybridized	2.7	-0.2	-0.1
10	Monomer	Not hybridized	2.7	-0.2	-0.1
12	Monomer	Not hybridized	2.6	-9.1	-3.7
13	Monomer	Not hybridized	2.6	-8.1	-3.3
8	Monomer	Not hybridized	2.1	-1.2	-0.5
18	Monomer	Not hybridized	2.1	-0.9	-0.4
29	Monomer	Not hybridized	1.4	-0.1	0.0
33	Monomer	Not hybridized	1.4	-0.1	-0.1
34	Monomer	Not hybridized	1.2	-1.1	-0.5
30	Monomer	Not hybridized	1.2	-1.9	-0.8

Table 7: Results of NUPACK computation, showing the equilibrium configuration for the strands listed in Table 6 (1-36). The maximum complex size was 2 strands, the temperature was 20°C, the concentration of all strands was 1 μM and 1 M sodium was used. All structures having a concentration greater than 0.01 μM are listed in this table. The yield is defined as a percentage, representing the concentration of the product divided by the reference concentration of 1 μM. All domains hybridize strongly with their intended targets.

2 Detailed experimental procedures: QCM-D experiments

2.1 General notes

- Apparatus used: Q-sense E4 machine, Biolin Scientific.
- Sensors: Gold-coated AT-cut quartz sensors (fundamental frequency 4.95MHz +/- 50kHz), Biolin Scientific, ref. no. QSX 301.
- Sensor cleaning: Before use, sensors were cleaned as follows, in accordance with the procedure described previously (see reference in manuscript). 10 minutes in UV-ozone cleaner, 10 minutes sonication in 2% Hellmanex III, 10 minutes sonication in ultrapure water (twice), drying with N_2 gas, 30 minutes in UV-ozone cleaner, 30 minutes soak in 100% ethanol, drying with N_2 gas.

The apparatus contains four flow modules, which means that up to four experiments can be run in parallel. Liquids were driven through the QCM-D apparatus using a peristaltic pump. The depth in solution to which an acoustic wave reaches is dependent on the frequency of the wave, and higher overtones probe only the region nearest to the surface. Data is shown for the thirteenth overtone, which is the highest frequency that can be observed with this system. The experiment was performed at 16°C, with a pump flow rate of 20μL/min. After sensors had been installed in the flow modules, the system was flushed with ultrapure water, followed by the experimental buffer, which was 1M NaCl in 1xTE (Tris-EDTA, pH8). Samples were then supplied and as the molecules bound to the surface, the frequency of the crystal decreased. The sections below provide step-by-step details of the workflow for each experiment performed.

2.1.1 Sequential strand displacement in a linear construct

The complex comprising CS and Block-3 was formed by mixing equimolar quantities of the two strands in a buffer of 1×TE with 1M NaCl and incubating the sample at room temperature for a period sufficient for hybridization. The CS/Block-3 complex was at a concentration of 300 nM, and all other strands were supplied at 750 nM. Between steps the sensor was washed with buffer for 4-5 minutes; throughout the experiment the buffer used was 1×TE with 1M NaCl. In the final step strand X* was applied, where X* is the reverse complement of strand X.

2.2 Strand displacement in a geometrically constrained structure ('Triangle' experiment)

Samples were prepared as follows. In all cases, the buffer was 1×TE with 1M NaCl. The final concentration is given.

- CS: 300nM
- T: 750nM
- S: 750nM ac-staple, 750nM bd-staple (note that the letter S is used later to denote something else)
- Control a: 750nM
- Control b: 750nM
- Control c: 750nM
- Control d: 750nM
- Additional controls (mixture): 750nM RC(ac-staple), 750nm RC(bd-staple)
- T*: 750nM

- F(T): 750nM T, 7.5μM ac-staple, 7.5μM bd-staple, anneal in thermal cycler from 95°C to 20°C at 1°C/min.
- F(T*) 750nM T*, 7.5μM RC(ac-staple), 7.5μM RC(bd-staple), anneal in thermal cycler from 95°C to 20°C at 1°C/min.

The QCM-D experiment was performed as described above. Samples were applied to the sensors as follows, where the approximate duration of each step of the experiment is shown in brackets, in minutes.

Fig. 3(b) top panel, Fig. 3(c) left panel: Buffer (17), CS (35), buffer (5), T (18), buffer (5), S (26), buffer (7), control a (10), buffer (7), control b (10), buffer (6), control c (10), buffer (8), control d(8), buffer (25), additional control (10), buffer (5), T* (until sample ran out)

Fig. 3(b) middle panel, Fig. 3(c) middle panel: Buffer (17), CS (35), buffer (28), F(T) (26), buffer (7), control a (10), buffer (7), control b (10), buffer (6), control c (10), buffer (8), control d (8), buffer (7), additional control (18), buffer (15), T*(until sample ran out)

Fig. 3(b) bottom panel, Fig. 3(c) right panel: Buffer (17), CS (35), buffer (28), F(T) (26), buffer (7), control a (10), buffer (7), control b (10), buffer (6), control c (10), buffer (8), control d (8), buffer (40), F(T*) (until sample ran out)

2.2.1 The rotary motor

Step 1: preparation of squares

The components were made as follows, by mixing the named strands in a buffer of 1×TE with 1M NaCl, and annealing from 95°C to 20°C in a thermocycler with a cooling rate of 1°C/min. The final concentration of each strand is given in brackets.

Definitions: □*=square; B=blocked; F=folded; UF=unfolded; S=functionalized for surface immobilization.*

BF□A = SquareA_1 (1μM), SquareA_2 (1μM), SquareA_3 (1μM), St96 (1μM), Spoke-A (1μM), St74 (5μM), St85 (5μM), SqBlockA (5μM)

F□A = SquareA_1 (1μM), SquareA_2 (1μM), SquareA_3 (1μM), St96 (1μM), Spoke-A (1μM), St74 (5μM), St85 (5μM)

BF□B = SquareB_1mmwA (1μM), SquareB_2 (1μM), SquareB_3 (1μM), Str69 (1μM), Spoke-B (1μM), Str47 (5μM), Str58 (5μM), SqBlockBmmwA (5μM)

F□B = SquareB_1mmwA (1μM), SquareB_2 (1μM), SquareB_3 (1μM), Str69 (1μM), Spoke-B (1μM), Str47 (5μM), Str58 (5μM)

UF□B = SquareB_1mmwA (1μM), SquareB_2 (1μM), SquareB_3 (1μM)

Step 2: preparation of surface attachment units

The following samples were prepared in the same buffer, and incubated at room temperature for approximately 30 minutes to allow the strands to hybridize.

T1 =1:1 mixture of CS and Bearing-A, final concentration 25μM

T2 =1:1 mixture of CS and Bearing-B, final concentration 25μM

Step 3: combination of squares and surface attachment units

Samples from step 1 were combined with samples from step 2 to form squares that could be immobilized on the gold surface, as follows. Mixtures were incubated at room temperature for a period sufficient to allow hybridization.

SBF□A = 1:25 (by volume), T1 :BF□A.

SF□A = 1:25 (by volume), T1 :F□A. (Like SBF□A, but without blocking strand.)

SBF□B = 1:25 (by volume), T2 :BF□B.

Note that 8 consecutive thymine nucleotides are left unpaired in the surface

attachment unit, to provide a flexible linker region that enables rotation to occur. If the linker were rigid, no rotation would be possible.

Step 4: mixing with MCH If the density of motors on the surface is too high, operation is impossible because they interact and/or collide. To avoid this, the samples from Step 3 were mixed with MCH (6-mercapto-1-hexanol), a thiolated molecule that will be immobilized at the same time as the motors. The original concentration of MCH was 1mM. In all cases, the final buffer was 1×TE with 1M NaCl.

Sample 1 = 1:1 (by volume), SF□A : MCH.

Sample 2 = 2:2:1 (by volume), SBF□A : SBF□B : MCH.

Sample 3 = As sample 1.

Sample 4 = 1:1 (by volume), SBF□A : MCH.

Sufficient time was allowed for the two halves of the motor to hybridize in sample 2.

Step 5: QCM-D experiments Freshly cleaned sensors were installed in the flow modules, which were then flushed with ultrapure water, followed by the experimental buffer, which was 1M NaCl/TE. The samples listed above were supplied, where sample X was used for flow module X. When a plateau was reached, the sensors were rinsed with buffer. The following solutions were then applied to the sensors:

Sensor 1: UF□B from Step 1, followed by buffer

Sensor 2 (consecutively): UnblockA (1μM), buffer (short rinse step), UnblockB_mmwA

Sensor 3: F□B from Step 1, followed by buffer

Sensor 4 (consecutively): UnblockA (1μM), buffer (short rinse step), UF□B from Step 1

In Fig. 4 of the paper, data is presented in this order: Sensor 1, 4, 3, 2.

2.3 Agarose gel electrophoresis: rotary motor, Fig. 4(c)

The samples were prepared as follows.

Step 1

The following mixture was incubated at room temperature for 30 mins in $1\times$TE with 1M NaCl. The final concentration of each strand is given in brackets.

SA = mixture of CS (1μM), Spoke-A (1μM) and Bearing-A (1μM)

Step 2

The following mixtures were annealed from 95°C to 20°C in a thermocycler with a cooling rate of 1°C/min.

F□A! = SquareA_1 (1μM), SquareA_2 (1μM), SquareA_3 (1μM), St96 (1μM), St74 (5μM), St85 (5μM)

F□B! = SquareB_1 (1μM), SquareB_2 (1μM), SquareB_3 (1μM), Str69 (1μM), Str47 (5μM), Str58 (5μM)

UF□B! = SquareB_1 (2.5μM), SquareB_2 (2.5μM), SquareB_3 (2.5μM)

o stands for original. The ! is used to distinguish the samples from those prepared for QCM-D, where the Spoke strands were added at this stage and SquareB_1mmwA was used in place of SquareB_1.

Extracts from these samples were prepared for gel electrophoresis and the gel was loaded as follows:

1. S
2. F□A!
3. F□B!
4. UF□B!

2.4 Polyacrylamide gel electrophoresis: rotary motor, Fig. 4(d)

Step 1

The following mixtures were annealed from 95°C to 20°C in a thermocycler with a cooling rate of 1°C/min. Unfolded square B, lane 5 - 50μL 2M NaCl/2xTE; 47μL ultrapure MilliQ water; 1μL of 100μM stock solution of each of: SquareB_1_mmwA, SquareB_2, SquareB_3.

Folded square B, lane 6 - 50μL 2M NaCl/2xTE; 31μL ultrapure MilliQ water; 1μL of 100μM stock solution of each of: SquareB_1_mmwA, SquareB_2, SquareB_3, Str69; 5μL of 100μM stock solution of each of: Str47, Str58, SqBlockB_mmwA.

Folded square A, lane 7 - 50μL 2M NaCl/2xTE; 31μL ultrapure MilliQ water; 1μL of 100μM stock solution of each of: SquareA_1, SquareA_2, SquareA_3, St96; 5μL of 100μM stock solution of each of: St74, St85, SqBlockA.

Step 2

Motor 'before': 20μL of folded square A was mixed with 20μL of folded square B, and the sample was incubated at room temperature for over 30 minutes.

Step 3

Motor 'after': A 20μL sample of the motor was extracted and the unblocking strands were added to a final concentration of approximately 4.5μM each. The sample was left at room temperature for over 30 minutes.

Step 4

Glycerol was added to a final concentration of approximately 12-13% and the gel was run as described in the Methods section of the main paper.
